# Synthesis and Characterization of Ceramide-Containing Liposomes as Membrane Models for Different T Cell Subpopulations

**DOI:** 10.3390/jfb13030111

**Published:** 2022-08-02

**Authors:** Sascha Eder, Claudia Hollmann, Putri Mandasari, Pia Wittmann, Fabian Schumacher, Burkhard Kleuser, Julian Fink, Jürgen Seibel, Jürgen Schneider-Schaulies, Christian Stigloher, Niklas Beyersdorf, Sofia Dembski

**Affiliations:** 1 Fraunhofer Institute for Silicate Research, ISC, 97082 Würzburg, Germany; eder.sascha@freenet.de (S.E.); pia.wittmann@stud-mail.uni-wuerzburg.de (P.W.); 2 Institute for Virology and Immunobiology, University of Würzburg, 97078 Würzburg, Germany; claudia.hollmann@gmx.net (C.H.); putri.mandasari@uni-wuerzburg.de (P.M.); jss@vim.uni-wuerzburg.de (J.S.-S.); niklas.beyersdorf@vim.uni-wuerzburg.de (N.B.); 3 Institute of Pharmacy, Freie Universität Berlin, 14195 Berlin, Germany; fabian.schumacher@fu-berlin.de (F.S.); burkhard.kleuser@fu-berlin.de (B.K.); 4 Institute of Organic Chemistry, University of Würzburg, 97074 Würzburg, Germany; julian.fink@uni-wuerzburg.de (J.F.); seibel@chemie.uni-wuerzburg.de (J.S.); 5 Imaging Core Facility of the Biocenter, University of Würzburg, 97074 Würzburg, Germany; christian.stigloher@uni-wuerzburg.de; 6 Department Tissue Engineering and Regenerative Medicine, University Hospital, 97070 Würzburg, Germany

**Keywords:** liposome, ceramide, cell membrane model

## Abstract

A fine balance of regulatory (T_reg_) and conventional CD4^+^ T cells (T_conv_) is required to prevent harmful immune responses, while at the same time ensuring the development of protective immunity against pathogens. As for many cellular processes, sphingolipid metabolism also crucially modulates the T_reg_/T_conv_ balance. However, our understanding of how sphingolipid metabolism is involved in T cell biology is still evolving and a better characterization of the tools at hand is required to advance the field. Therefore, we established a reductionist liposomal membrane model system to imitate the plasma membrane of mouse T_reg_ and T_conv_ with regards to their ceramide content. We found that the capacity of membranes to incorporate externally added azide-functionalized ceramide positively correlated with the ceramide content of the liposomes. Moreover, we studied the impact of the different liposomal preparations on primary mouse splenocytes in vitro. The addition of liposomes to resting, but not activated, splenocytes maintained viability with liposomes containing high amounts of C_16_-ceramide being most efficient. Our data thus suggest that differences in ceramide post-incorporation into T_reg_ and T_conv_ reflect differences in the ceramide content of cellular membranes.

## 1. Introduction

Besides phosphoglycerides and cholesterol (CHOL), bioactive sphingolipids are the main structural components of the eukaryotic cell membrane [1]. In particular, ceramide, which serves as a building hub for complex sphingolipids, influences the biophysical properties of the plasma membrane and organizes the formation of membrane microdomains that are crucial for receptor clustering [2]. While these processes fundamentally impact the biology of many cell types, sphingolipid metabolism in T cells is of particular importance due to their broad involvement in diseases as different as cancer and infections, including COVID-19, and their accessibility to therapeutic intervention [3].

In T cells, ceramide-enriched microdomains are known to modulate signaling of various cell surface receptors including the T cell receptor complex [4]. Moreover, T cell subpopulations differ regarding their ceramide content. For instance, CD4^+^ Foxp3^+^ regulatory T cells (T_reg_) protecting the body from autoimmunity and immunopathology have been shown to have a much higher ceramide content than CD4^+^ Foxp3^−^ conventional (T_conv_) T helper cells [5,6]. In humans, the incorporation of clickable ceramide is positively correlated with effector/memory differentiation of both T_reg_ and T_conv_ [7]. While the determination of cellular ceramide content using mass spectrometry reflects ceramide content at the population level, minimally modified ceramides suitable for click chemistry allow the ceramide content to be determined at the single-cell level using flow cytometry, fluorescence, or confocal microscopy [8,9]. After the incubation of cells with azide-functionalized ceramide, the incorporated ceramide can be visualized with reagents such as the Click-IT Alexa Fluor 488 DIBO Alkyne (DIBO Alexa Fluor 488) [10]. Therefore, the detection of incorporated azide-functionalized ceramide can easily be combined with multi-color flow cytometry, covering more than ten parameters in parallel. The detection of incorporated ceramide in combination with other markers can thus not only be used for research, but potentially also for diagnostic purposes. Currently, it is, however, unclear what varying degrees of incorporation of clickable ceramides actually stand for, i.e., whether they reflect cellular ceramide content or whether the composition of lipid microdomains is the primary determinant of clickable ceramide incorporation.

Over the past decade, several reconstituted membrane models, e.g., planar membranes, as well as solid-supported membrane and liposomes, have been evaluated [11]. Liposomes are “soft” biocompatible, biodegradable, and non-immunogenic nanostructures [12]. These unique features and the possibility of adding targeting moieties to their surfaces make liposomes very attractive candidates as drug delivery vehicles [13]. In addition, due to their phospholipid bilayer, size, and morphology, liposomes are a suitable cell model system [14]. Liposomes have been used, e.g., for the investigation of permeation processes [15] and the estimation of the interaction of new drugs with cell membranes [16]. By integrating various proteins or CHOL into the lipid bilayer, a liposome-based membrane model can mimic different cells and allow, e.g., the measurement of the activity of transmembrane proteins or protein complexes [17]. Through variations of the lipid formulation, vesicle size, or surface charge, the effects of various biophysical parameters on the interaction between liposomal bilayers and low-molecular-weight substances can be investigated [18]. Furthermore, liposomes are suitable for the investigation of membrane fusion processes [19], as well as the transfer of membrane components [20] or membrane protein properties [21]. More specifically, the liposome-based model is suitable for the characterization of domain formation [22] and the investigation of the influence of ceramides on the structure of membrane microdomains [23].

Membranes and cellular content are transferred from one cell to another following the release of exosomes. The activity of the neutral sphingomyelinase 2 (Nsm2) is mandatory to enable cells to form and release exosomes [22]. With its localization in the inner leaflet of the cell membrane, ceramide generation by Nsm2 favors its outward protrusion and thus exosomal release. As a consequence, exosomes contain about three times more ceramide than cellular membranes [24]. For lymphocytes, it has been shown that activation greatly increases exosomal release, with T_reg_ being about twice as efficient as conventional CD4^+^ T cells and B cells [25].

In this study, a reductionist liposomal membrane [24] model system was established to imitate the plasma membrane of mouse T_reg_ and T_conv_ with regard to their ceramide content. This model system was used to investigate whether the initial ceramide content in the membrane determines the capacity of the post-incorporation of ceramides into the lipid layer. In addition, we studied the impact of liposomes with different ceramide contents on the viability of primary mouse splenocytes in vitro.

## 2. Materials and Methods

### 2.1. Animals

C57BL/6 mice were bred under specific pathogen-free conditions and in accordance with German law in the animal facility of the Institute for Virology and Immunobiology of the University of Würzburg. In compliance with the 3R principles, particularly a reduction in animals used for scientific purposes, mice were used which were phenotypically wildtype, i.e., were Thy1.1-congenic or carried loxP sites without Cre recombinase expression or expressed a tamoxifen-inducible Cre recombinase without, however, receiving tamoxifen. The license to breed and house mice at the Institute for Virology and Immunobiology of the University of Würzburg for scientific purposes was obtained from the City of Würzburg (FB VVL 568/300-1870/13) on 29 June 2016.

### 2.2. Materials

1-palmitoyl-2-oleoyl-sn-glycero-3-phosphocholine (POPC) > 99% was purchased from Avanti Polar Lipids (Alabaster, AL, USA), cholesterol (CHOL) from Sigma-Aldrich (Taufkirchen, DE, Germany), and DIBO Alexa Fluor 488 from Invitrogen (Waltham, MA, USA). Commercially available chemical reagents, purchased from Sigma-Aldrich (Taufkirchen, DE, Germany), Alfa Aesar (Ward Hill, MA, USA), TCI (Eschborn, Germany), and Acros Organics (Fair Lawn, NJ, USA), were used as received without further purification. All solvents were distilled before usage and dried when needed using standard procedures. The moisture-sensitive reactions were carried out under nitrogen atmosphere. Analytical thin-layer chromatography (TLC) was performed using silica gel pre-coated aluminum plates with a thickness of 0.2 mm. The compounds were visualized with a potassium permanganate stain solution (1.50 g of KMnO_4_, 10.0 g of K_2_CO_3_, 100 mg of NaOH, and 200 mL of H_2_O). Liquid column chromatography purification was performed with silica gel 60 (40–63 μm mesh) from Macherey–Nagel (Düren, DE).

### 2.3. Chemical Synthesis of Clickable Ceramide Analogues

The clickable ceramide derivatives ω-N_3_-C_6_-Cer (4) and ω-N_3_-C_16_-Cer (5) were synthesized by HBTU-mediated amide coupling of sphingosine (1) with the corresponding azido-acids 2 and 3 (Scheme S1), which have been prepared according to literature [9].

#### 2.3.1. 6-Azido-*N*-((2S,3*R,E*)-1,3-dihydroxyoctadec-4-en-2-yl)hexanamide/ω-N_3_-C_6_-Cer (4)

To a solution of 6-azidohexanoic acid (2) (26.2 mg, 167 μmol, 1.00 eq.) in dry DMF (5 mL), DIPEA (87.2 μL, 501 μmol, 3.00 eq.) and HBTU (76.0 mg, 200 μmol, 1.20 eq.) were added at 0 °C. After stirring at this temperature for 15 min, sphingosine (1) (50.0 mg, 167 μmol, 1.00 eq.) and dry DMF (4 mL) were added in one portion. The ice bath was removed and the reaction mixture was stirred at RT for 2 h. Saturated aq. NH_4_Cl solution (50 mL) and H_2_O (5 mL) were added, before then being extracted with CH_2_Cl_2_ (6 × 25 mL). The combined organic phases were washed with brine (20 mL), dried (MgSO_4_), and concentrated under reduced pressure. The residue was purified twice by column chromatography on silica gel (1. CHCl_3_/MeOH 20:1, 2. CHCl_3_/MeOH 40:1) to give (4) (56.2 mg, 128 μmol, 77%) as a colorless waxy solid.

^1^H-NMR (CDCl_3_, 400 MHz): δ = 0.88 (t, ^3^J_18,17_ = 6.9 Hz, 3H, H-18), 1.25–1.31 (m, 20H, H-8–17), 1.35–1.46 (m, 4H, H-7, H-4′), 1.59–1.72 (m, 4H, H-5′, H-3′), 2.05 (app br q, J = 7.1 Hz, 2H, H-6), 2.25 (t, ^3^J_2′,3′_ = 7.5 Hz, 2H, H-2′), 2.47 (br s, 2H, 2 × OH), 3.28 (t, ^3^J_6′,5′_ = 6.8 Hz, 2H, H-6′), 3.70 (dd, ^2^J_1a,1b_ = 11.3 Hz, ^3^J_1a,2_ = 3.3 Hz, 1H, H-1a), 3.88–3.93 (m, 1H, H-2), 3.96 (dd, ^2^J_1b,1a_ = 11.3 Hz, ^3^J_1b,2_ = 3.7 Hz, 1H, H-1b), 4.31–4.34 (m, 1H, H-3), 5.53 (ddt, ^3^J_4,5_ = 15.4 Hz, ^3^J_4,3_ = 6.4 Hz, ^4^J_4,6_ = 1.3 Hz 1H, H-4), 5.79 (dtd, ^3^J_5,4_ = 15.4 Hz, ^3^J_5,6_ = 6.8 Hz, ^4^J_5,3_ = 1.0 Hz, 1H, H-5), 6.30 (d, ^3^J_NH,2_ = 7.5 Hz, 1H, NH) ppm;

^13^C-NMR (CDCl_3_, 150 MHz): δ = 14.3 (C-18), 22.8 (C-17), 25.3 (C-3′), 26.4 (C-4′), 28.7 (C-5′), 29.3 (C-7), 29.4, 29.5, 29.6, 29.8, 29.8, 29.8, 29.8 (together 8C, C-8–15), 32.1 (C-16), 32.4 (C-6), 36.6 (C-2′), 51.4 (C-6′), 54.5 (C-2), 62.5 (C-1), 74.8 (C-3), 128.9 (C-4), 134.4 (C-5), 173.5 (C-1′) ppm;

HRMS (ESI^+^): *m/z* calcd. for C_24_H_46_N_4_NaO_3_ [M+Na]^+^: 461.34621, found: 461.34596 (|Δ*m/z*| = 0.54 ppm). The measured spectroscopic data are in agreement with previously reported data [9].

#### 2.3.2. 16-Azido-*N*-((2S,3*R,E*)-1,3-dihydroxyoctadec-4-en-2-yl)hexadecanamide/ω-N_3_-C_16_-Cer (5)

To a solution of 16-azidohexadecanoic acid (3) (49.7 mg, 167 μmol, 1.00 eq.) in dry DMF (5 mL), DIPEA (87.2 μL, 501 μmol, 3.00 eq.) and HBTU (76.0 mg, 200 μmol, 1.20 eq.) were added at 0 °C. After stirring at this temperature for 15 min, sphingosine (1) (50.0 mg, 167 μmol, 1.00 eq.) and dry DMF (4 mL) were added in one portion. The ice bath was removed and the reaction mixture was stirred at RT for 2 h. Saturated aq. NH_4_Cl solution (50 mL) and H_2_O (5 mL) were added, before then being extracted with CH_2_Cl_2_ (6 × 25 mL). The combined organic phases were washed with brine (20 mL), dried (MgSO_4_), and concentrated under reduced pressure. The residue was purified using column chromatography on silica gel (CHCl_3_/MeOH 40:1) to give (5) (95.4 mg, 165 μmol, 99%) as a colorless solid.

^1^H-NMR (CDCl_3_, 400 MHz): δ = 0.81 (t, ^3^J_18,17_ = 6.9 Hz, 3H, H-18), 1.19–1.31 (m, 44H, H-7–17, H-4′–14′), 1.49–1.61 (m, 4H, H-3′, H-15′), 1.99 (app br q, J = 7.1 Hz, 2H, H-6), 2.16 (t, ^3^J_2′,3′_ = 7.6 Hz, 2H, H-2′), 2.60 (br s, 2H, 2× OH), 3.19 (t, ^3^J_16′,15′_ = 7.0 Hz, 2H, H-16′), 3.64 (dd, ^2^J_1a,1b_ = 11.2 Hz, ^3^J_1a,2_ = 3.2 Hz, 1H, H-1a), 3.82–3.86 (m, 1H, H-2), 3.90 (dd, ^2^J_1b,1a_ = 11.2 Hz, ^3^J_1b,2_ = 3.7 Hz, 1H, H-1b), 4.24–4.27 (m, 1H, H-3), 5.46 (ddt, ^3^J_4,5_ = 15.4 Hz, ^3^J_4,3_ = 6.4 Hz, ^4^J_4,6_ = 1.3 Hz, 1H, H-4), 5.72 (dtd, ^3^J_5,4_ = 15.4 Hz, ^3^J_5,6_ = 6.7 Hz, ^4^J_5,3_ = 1.0 Hz, 1H, H-5), 6.17 (d, ^3^J_NH,2_ = 7.4 Hz, 1H, NH) ppm;

^13^C-NMR (CDCl_3_, 100 MHz): δ = 14.3 (C-18), 22.8 (C-17), 25.9 (C-3′), 26.9 (C-14′), 29.0 (C-15′), 29.3, 29.3, 29.4, 29.4, 29.5, 29.6, 29.6, 29.7, 29.8, 29.8, 29.8 (together 19C, C-7–15, C-4′–13′), 32.1 (C-16), 32.4 (C-6), 37.0 (C-2′), 51.6 (C-16′), 54.6 (C-2), 62.6 (C-1), 74.8 (C-3), 128.9 (C-4), 134.4 (C-5), 174.1 (C-1′) ppm;

HRMS (ESI^+^): *m/z* calcd. for C_34_H_66_N_4_NaO_3_ [M+Na]^+^: 601.50271, found: 601.50316 (|Δ*m/z*| = 0.75 ppm). The measured spectroscopic data are in agreement with previously reported data [9].

### 2.4. General Liposome Preparation Procedure

For the synthesis of liposomes, several stock lipid solutions were prepared. Table 1 gives an overview of the lipids used for liposome preparation. The concentration of the stock solutions in CHCl_3_ was 5 mM. In order to investigate the post-synthetic incorporation of the ceramide derivatives, additional stock solutions of the azide-modified ceramides in EtOH with a concentration of 25 mM were prepared. To dissolve ω-N_3_-C_16_-Cer, its ethanolic solution was additionally heated up to 50 °C. All stock solutions were stored at −25 °C.

Ceramide-free and ceramide-containing liposomes were prepared using a lipid film hydration method following freeze-thawing. For the multi-component lipid formulation, the stock solutions in CHCl_3_ were combined in a certain vol% ratio and evaporated using a rotary evaporator. The total volume before evaporation was always 1 mL. The resulting dry lipid film was additionally dried for 15 min under a pressure of 10–18 mbar and rehydrated with 1 mL of PBS. Ceramide-free and C_6_-ceramide-containing lipid formulations (C_6_-Cer, ω-N_3_-C_6_-Cer) were incubated for 1 h at 30 °C in a water bath. C_16_-ceramide-containing formulations (C_16_-Cer, ω-N_3_-C_16_-Cer) were incubated for 30 min at 50 °C in a water bath. The resulting mixture was homogenized for 30 s at 3000 rpm using a vortexer, freeze-thawed five times, and extruded once through a PC membrane with a pore size of 400 nm and 15 times with a pore size of 100 nm. Resulting liposomes were purified by a GPC. The chromatography column was equilibrated with 10 mL of PBS. The liposomal dispersion was diluted up to a total volume of 2.5 mL of PBS and applied to the column. The liposomes were eluted with 2 mL of PBS. For the transmission electron microscopy (TEM), liposomes were eluted with 2 mL of HEPES buffer.

### 2.5. Post-Insertion of Azide-Modified Ceramides and Click Reaction

Next, 25 mM stock solutions of azide-modified ceramides in EtOH (see Table 1) were diluted 1:1000 with PBS. The final concentration of all solutions was 25 µM. In each case, 500 µL of ceramide-modified liposome dispersion was incubated with 500 µL of diluted azide-modified ceramide solution for 12 h at 25 °C (ω-N_3_-C_6_-Cer) or 65 °C (ω-N_3_-C_16_-Cer) and 300 rpm in a thermoshaker. As a control, liposomes were incubated with 500 μL of EtOH:PBS 1:1000 mixture at 25 and 65 °C, respectively. After incubation, the dispersions were purified by GPC (see section before). Subsequently, 80 μL of the DIBO Alexa Fluor 488 (25 μM) was added. All dispersions were incubated for 12 h at RT and 300 rpm in a thermoshaker and purified by GPC again.

To quantify the amount of integrated azide-modified ceramides, 1 mL of each liposome dispersion was incubated with 80 μL of DIBO Alexa Fluor 488 for 12 h at RT. Liposomes were extruded through a 100 nm PC membrane and purified by GPC.

Primary mouse lymph node cells (1 × 10^7^) from wildtype C57BL/6J mice (bred at the Institute for Virology and Immunobiology, see below) were incubated for 30 min at room temperature with N_3_-C_6_-Cer (25 μM) in HBSS medium containing Pluronic F-127 (20% in DMSO). Controls were incubated in HBSS medium only. After washing, Alexa Fluor 488 DIBO Alkyne (20 μM; Life Technologies) was added for 10 min to react with incorporated N_3_-C_6_-Cer in a click reaction. Anti-CD25 APC was added in parallel to Alexa Fluor 488 DIBO Alkyne. After three washings with HBSS, cells were transferred to 12-well slides for microscopy. For confocal microscopy, an LSM 780 confocal microscope from Zeiss (Jena, DE) equipped with a 40× Plan-Apochromat oil objective and 404 nm, 488 nm, and 633 nm lasers were used. Pictures were acquired and processed using ZEN2012 software (Zeiss). For FACS analysis, the cells were stained with anti-CD4 brilliant violet 421, anti-CD25 APC, and anti-CD44 PE. To discriminate between dead cells which very readily incorporate Alexa Fluor 488 DIBO Alkyne and live cells, the cells were incubated with Viability Dye eFluor ™ 780 from eBioscience (Carlsbad, CA).

### 2.6. Nuclear Magnetic Resonance (NMR)

NMR spectra were recorded on a Bruker Avance III HD 400 or 600 at 295 K. Chemical shifts (δ) are given in ppm with respect to the solvent residual proton signals (δ(CDCl_3_) = 7.26 ppm) for ^1^H or the resonance signal (δ(CDCl3) = 77.16 ppm) for ^13^C. Coupling constants (J) are reported in Hertz (Hz) and the multiplicity is abbreviated as s (singlet), d (doublet), t (triplet), m (multiplet), dd (doublet of doublets), app br q (apparent broad quartet), etc. Signal assignment was performed with additional information of DEPT135, (^1^H,^1^H)-COSY, (^1^H,^13^C)-HSQC, and (^1^H,^13^C)-HMBC. Atom numbers do not refer to the IUPAC nomenclature.

### 2.7. High Resolution Mass Spectrometry (HRMS)

HRMS was performed with a Bruker Daltonics micrOTOF-Q III (electrospray ionization, ESI) instrument.

### 2.8. Transmission Electron Microscopy (TEM)

TEM images of the liposomes were obtained on a JEM-1400Flash microscope (JEOL, Akishima, Japan) equipped with a Matataki Flash camera system. For negative contrast, carbon-coated 300-mesh copper grids were processed by a glow discharge. Subsequently, 20 μL of the liposome dispersion were dripped onto a grid. For the better contrast, 7 µL of 2% aq. uranyl acetate (UA) was added to the copper grid for 5 min. UA solution was removed with a filter paper and the grid was dried on the air for 10 min. TEM images were analyzed with the software Image J [26]. In total, 100 liposomes of each sample were analyzed (Figure S7).

### 2.9. Dynamic Light Scattering (DLS)

For the detection of hydrodynamic diameter, the liposome dispersions were diluted 1:50 with PBS. Then, 1.5 mL of diluted solution was transferred to a polystyrene cuvette. DLS measurement was performed on a Zetasizer ZS (Malvern Instruments, Malvern, UK).

### 2.10. Nanoparticle Tracking Analysis (NTA)

The particle size, size distribution, and particle concentration of liposomes were determined by NTA on a NanoSight NS 300 device from NanoSight (Malvern Panalytical, Malvern, UK). For the NTA measurement, the liposome dispersions were diluted 1:200 with PBS. The examined volume was 1 mL. The analysis of measurement data was performed with the software NanoSight NTA 3.2.

### 2.11. Quantification of Liposomal Ceramides by HPLC-MS/MS

Liposome samples were diluted 1:100 (*v*:*v*) with water. Dilutions were processed in triplicates as follows: 800 µL of water and 110 µL of 10× Baker buffer (300 mM citric acid, 400 mM disodium hydrogen phosphate, pH 3.0) were added to a 200 µL sample. For lipid extraction, 2 mL of 1-butanol and 1 mL of water-saturated 1-butanol were added. The extraction solvent contained 50 pmol C_17_-ceramide (C_17_-Cer) (Avanti Polar Lipids, Alabaster, AL, USA) as an internal standard. Extraction was facilitated by intensive vortexing (1500 rpm) for 10 min at RT. Afterwards, samples were centrifuged for 5 min at 2200× *g* (4 °C). The upper organic phase was dried under reduced pressure using a Savant SpeedVac concentrator (Thermo Fisher Scientific, Dreieich, Germany). Dried residues were reconstituted in 200 μL of acetonitrile/MeOH/H_2_O (47.5:47.5:5 (*v*:*v*:*v*), 0.1% formic acid) and subjected to HPLC-MS/MS ceramide quantification. Chromatographic separation was achieved on a 1290 Infinity II HPLC (Agilent Technologies, Waldbronn, Germany), equipped with a Poroshell 120 EC-C8 column (3.0 × 150 mm, 2.7 µm; Agilent Technologies), guarded by a pre-column of identical material. MS/MS analysis was carried out using a 6495 triple-quadrupole mass spectrometer (Agilent Technologies) operating in the positive electrospray ionization mode (ESI+) [27]. The following mass transitions were recorded for analysis of liposomal ceramides (collision energies (CE) in parentheses): *m/z* 380.4 → 264.3 (25 eV, quantifier) and *m/z* 380.4 → 282.3 (15 eV) for C_6_-Cer, *m/z* 520.5 → 264.3 (25 eV, quantifier) and *m/z* 520.5 → 282.3 (15 eV) for C_16_-Cer, and *m/z* 534.5 → 264.3 (25 eV) for the internal standard C_17_-Cer. Data processing and quantification were performed with MassHunter Software (Agilent Technologies).

### 2.12. Fluorescence Spectroscopy

The emission spectra were recorded with the Infinite M1000 Pro microplate reader (TECAN, Männedorf, Switzerland). Following this, 200 μL of the undiluted dispersions was transferred into a black 96-well microplate. Spectra were recorded in the range of 500 to 650 nm at the excitation wavelength of 488 nm. The measurement evaluation was carried out with the software TECAN I-Control Version 1.12.4.0.

### 2.13. Culture and Analysis of Mouse Splenocytes

In line with the 3R principle, C57BL/6J mice, i.e., phenotypically wildtype mice (‘wrong genotype’) from different strains of genetically modified mice, were used to obtain cells for our in vitro experiments. Mice were bred and maintained in the specific pathogen-free animal facility of the Institute of Virology and Immunobiology of the University of Würzburg in accordance with German law. Animals used for the experiments were aged between 23 and 31 weeks.

To obtain a single-cell suspension, the spleen was extracted and homogenized through 70 µm cell strainers in BSS/BSA. After centrifugation (1600 rpm, 5 min), erythrocyte lysis was performed to the cell suspension while vortexing using equal volumes of NaCl (1.8%) and ddH_2_O, followed by 10 min incubation on ice. The supernatant was washed in BSS/BSA and the live cells were counted using Trypan blue dead cell exclusion.

Next, 2 × 10^5^ cells of splenocytes were incubated in a RPMI 1640 medium in the presence of Con A (5 µg/mL) with or without different liposomes with various C_16_-Cer (C_16_ high, C_16_ low, and C_16_ free) and different liposome to cell ratio (10:1 to 10,000:1) in 96-well round-bottom plate for 48 h at 37 °C. As a negative control, cells were cultured alone without Con A or liposome.

For FACS staining, cultured cells were initially washed with FACS buffer. Cells were then blocked with anti-CD16/anti-CD32 (clone 2.4G2; Fc blocker) for 15 min at 4 °C and the cells were incubated with the respective fluorochrome-labelled antibodies (extracellular markers: CD4, CD8a, B220/CD45R, CD25, CD69, and Viability Dye) in the dark for another 15 min at 4 °C. After washing with PBS, cells were fixated and permeabilized using the fixation–permeabilization buffer (eBioscience) according to the manufacturer’s protocol. Intracellular staining for Ki-67 was conducted for 45 min at RT. Cells were washed twice with the permeabilization buffer (eBioscience) and the FACS buffer, respectively, before flow cytometry measurement. The antibodies used for the FACS analysis were anti-CD4 Pacific Blue (clone RM4-5), anti-CD8a Alexa Fluor 700 (clone 53-6.7), anti-B220/CD45R PE-Cy5 (clone RA3-6B2), anti-CD25 FITC (clone PC61), anti-CD69 PE (clone H1.2F3), anti-Ki-67 Alexa Fluor 647 (clone B56), and Viability Dye eFluor 780.

## 3. Results

### 3.1. T_reg_ and Effector/Memory T_conv_ of Mice Incorporated More Ceramide into Their Cell Membrane Than Naïve T_conv_

**Figure 1:**
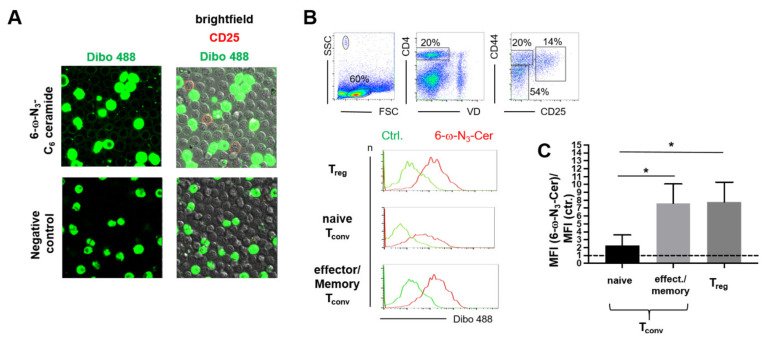
T_reg_ and effector/memory T_conv_ incorporate more ceramide than naive T_conv_. (**A**) Purified CD4^+^ lymph node cells of C57BL/6 mice were incubated with ω-azide-functionalized C_6_-ceramide and then reacted with DIBO 488 in a click reaction (green, top row). CD25 was used as a marker for T_reg_ (red). The figure shows confocal microscopy images of DIBO 488-labeled cells (left column) and the overlay of DIBO 488, CD25, and the light microscopy images (right column). Cells with a brightly green cytoplasm are dead cells. Lower row: Negative controls without ω-azide-functionalized C_6_-ceramide and anti-CD25 mAb incubation. Original magnification: 40× plan apochromat objective lenses. The program Zen2012 for confocal microscopy was used to analyze and generate the images. (**B**) Gating strategy to detect T_reg_, naïve, and effector/memory T_conv_ using the markers CD25 and CD44 (dot plots). The histogram overlays show representative stainings for DIBO 488 after incubation with (red line) or without ω-N_3_-C_6_-Cer incubation (green line). (**C**) Summary graph depicting C_6_-ceramide incorporation into T_reg_, CD44^-^ CD25^-^ naïve, and CD44^+^ CD25^-^ effector/memory T_conv_. Means + SD of *n* = 4 mice are shown. The dashed line indicates no specific binding of the ω-N_3_-C_6_-Cer. An unpaired two-sided t-test was used to assess statistical significance (* *p* < 0.05).

For mouse T_reg_, it has been shown that their membranes contain more ceramide than T_conv_ [5,6]. In line with this, mouse T_reg_ as well as effector/memory cells such as T_conv_ incorporated more of the di-4-ANEPPDHQ (ANE) dye into the cell membrane, indicating a lower order of lipids to which a higher ceramide content contributes [6,28,29]. For human T_reg_ and T_conv_, there was a positive correlation between the incorporation of the ANE dye and the cumulation of clickable ceramide in the cell membrane [7]. To test whether this also holds true for mouse T_reg_, we analyzed the incorporation of ω-N_3_-functionalized C_6_-ceramide into the cell membrane using confocal microscopy (Figure 1A). Flow cytometric analysis of ceramide incorporation comparing naïve T_conv_, effector/memory T_conv_, and T_reg_ cells further showed that both T_reg_ and effector/memory T_conv_ cells incorporated more ceramide than naïve T_conv_ (Figure 1B,C). Therefore, for both human and mouse CD4^+^ T cells, higher ANE and C_6_-ceramide incorporation were positively correlated.

### 3.2. Generation of Liposomes with High and Low Ceramide Content

To investigate whether the initial ceramide content in the membrane determines the capacity of post-incorporation of ceramides into the lipid layer, azide-modified ceramides (ω-N_3_-Cer) were synthesized and used for the preparation of liposomes. This enabled the covalent bonding of fluorescent dye molecules using a bio-orthogonal click reaction and subsequent fluorescence spectroscopic and microscopic analyses [30]. To generate liposomal membranes as reductionist model systems for T_reg_ and T_conv_, liposomes with different ceramide contents and carbon chain lengths (C_6_-Cer and C_16_-Cer) were prepared using the film method followed by freeze–thaw processes and extrusion through a polycarbonate (PC) membrane [31]. Lipid vesicles with the size of 100 nm were made of 1-palmitoyl-2-oleoyl-sn-glycero-3-phosphocholine (POPC) and CHOL. The amount of CHOL was varied between 10 and 50 volume percent (vol%). The content of POPC was either equal or higher than the ceramide content but the amount of ceramide was, under all conditions, at least 20 vol%. As a reference, ceramide-free liposomes of the composition POPC:CHOL = 75:25 vol% were synthesized. Stable ceramide-modified liposomes (C_6_-Cer, C_16_-Cer) with a size of about 100 nm and narrow particle size distribution were obtained in the composition of Cer:POPC:CHOL = 30:45:25 vol%. Therefore, we used this composition as a membrane model for ceramide-rich mouse T_reg_. For relatively ceramide-poor T_conv_, a lipid composition of Cer:POPC:CHOL = 15:35:50 vol% was used [6]. The tested liposome compositions and determined particle sizes and concentrations are shown in Table 2.

**Figure 2:**
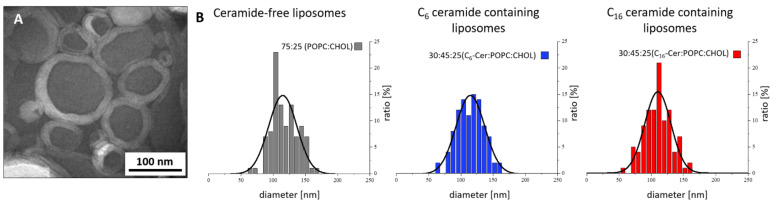
Representative TEM micrograph of liposomes prepared by the film method (**A**); evaluation of TEM micrographs (**B**).

Regardless of the ceramide content, all liposome samples had a spherical unilamellar structure and showed no significant morphological differences (Figure 2).

High-performance liquid chromatography–tandem mass spectrometry (HPLC-MS/MS) was applied to quantify the exact amount of ceramide incorporated into the membrane of the liposomes. The amounts of ceramide measured per sample were normalized to the size of the particles and their concentration as determined by NTA (Table 3).

**Figure 3:**
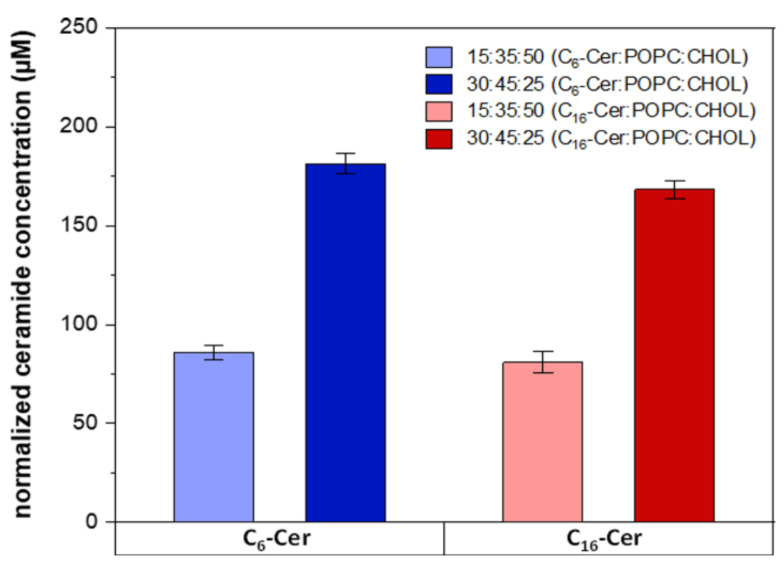
Ceramide concentration in the liposomes determined by HPLC-MS/MS. Means ± SD (*n* = 3).

The normalized ceramide contents showed that the quantity of ceramide incorporated into the liposomal membranes matched the vol% ratios used during liposome synthesis (Figure 3).

**Figure 4:**
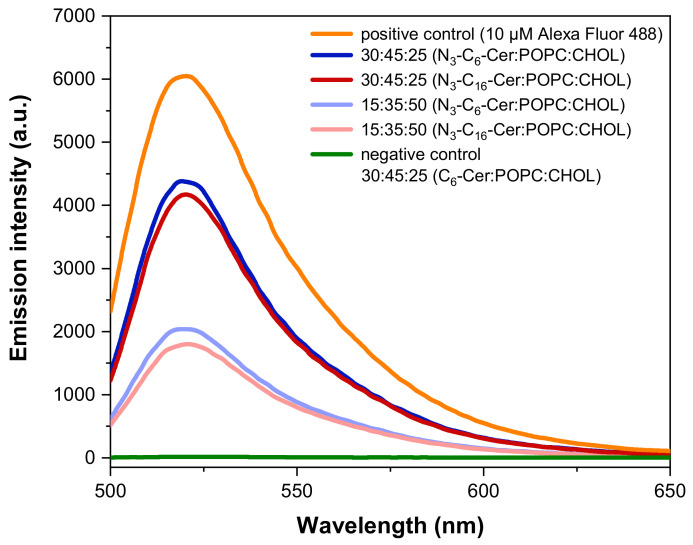
Emission spectra of ω-N_3_-C_6_-Cer- and ω-N_3_-C_16_-Cer-containing liposomes after the click reaction with DIBO Alexa Fluor 488 at λ_exc_ = 488 nm (*n* = 1). The experiment was repeated with similar results.

To determine whether differences in the ceramide content can also be visualized using azide-modified ceramides, liposomes containing azide-modified ceramide were prepared. For the synthesis of these liposomes, the same ratios of azide-modified ceramides, POPC, and CHOL were used as for non-functionalized ceramides. Subsequently, the fluorescent dye DIBO Alexa Fluor 488 was covalently bound to the azido-ceramide-modified liposomes via a click reaction. Free dye was removed by gel permeation chromatography (GPC). The binding capacity was analyzed by fluorescence spectroscopy in comparison to pure dye (10 µM solution in PBS) and a liposome preparation without azide-modified ceramide. The fluorescence spectra of liposomes after the click reaction with the fluorescent dye are shown in Figure 4.

The pure dye solution had the strongest emission intensity, while the samples without azide-modified ceramide showed almost no signal. This confirms that there is no unspecific binding of DIBO Alexa Fluor 488 to the liposomal membrane. Furthermore, both ceramide-containing liposome samples synthesized with a vol% ratio of 30:45:25 (ω-N_3_-C_6_/C_16_-Cer:POPC:CHOL) showed about twice the fluorescence intensity signal as the vesicles prepared with a vol% ratio of 15:35:50 (ω-N_3_-C_6_/C_16_-Cer:POPC:CHOL). At the same N_3_-Cer:POPC:CHOL ratio, there were no significant differences between the samples with different chain lengths of the fatty acid contained in the ceramide. This applied to vesicles for which 15 and 30 vol% of azide-modified ceramide had been used. However, it could be seen that the ω-N_3_-C_6_-Cer-containing samples had slightly higher intensities compared to the ω-N_3_-C_16_-Cer samples, which is in line with the quantifications obtained by HPLC-MS/MS (Figure 3).

### 3.3. Ceramide Content of Liposomes Is Positively Correlated with Incorporation of Clickable Ceramides

**Figure 5:**
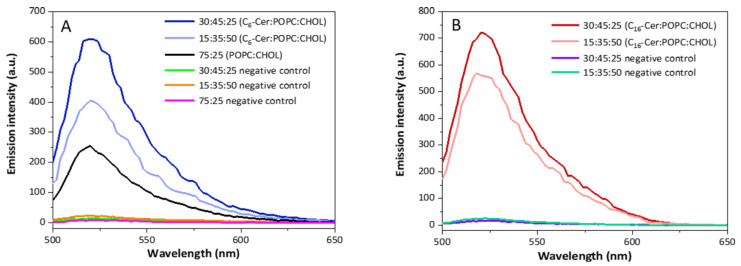
Emission spectra (λ_exc_ = 488 nm) of (**A**) C_6_-Cer- and (**B**) C_16_-Cer-containing liposomes after post-incorporation of azido-modified ceramides and click reaction with DIBO Alexa Fluor 488 (*n* = 1). The experiment was repeated with similar results.

Different amounts of ceramide were used for synthesis, which led to the generation of liposomes with a two-fold difference in ceramide content as intended. We used these liposomes to investigate the influence of ceramide content on the incorporation of clickable ceramides. For this purpose, all liposome formulations (ceramide-free and ceramide-containing) were incubated with azide-modified ceramide compounds (Table 2). Liposomes containing C_6_-Cer were incubated with ω-N_3_-C_6_-Cer and samples containing C_16_-Cer with ω-N_3_-C_16_-Cer, respectively. As a negative control, liposome samples were incubated with an EtOH:PBS mixture. Subsequently, all liposome samples were incubated with the fluorescent dye DIBO Alexa Fluor 488. The fluorescence spectra of liposomes after the click reaction with the fluorescent dye are shown in Figure 5.

The emission intensities were normalized to the particle concentration determined by the NTA (Table 2). The emission spectra showed that ceramide-containing liposomes incorporated more azido-modified ceramides than ceramide-free samples.

**Figure 6:**
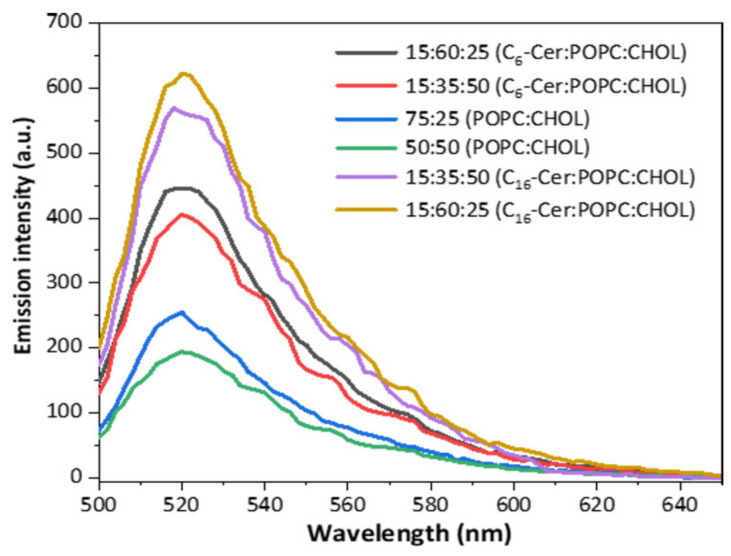
Emission spectra of ceramide-free and C_6_-Cer- and C_16_-Cer-containing liposomes with different CHOL content after post-insertion of azido-modified ceramides and click reaction with DIBO Alexa Fluor 488 at λ_exc_ = 488 nm (*n* = 1).

Additionally, the influence of the CHOL content in the liposomal membrane on the ceramide post-incorporation was investigated. To this end, liposomes with different amounts of CHOL, e.g., 50:50 vol% (POPC:CHOL), 15:60:25 vol% (C_6_-Cer:POPC:CHOL) and 15:60:25 vol% (C_16_-Cer:POPC:CHOL), were synthesized, incubated with azido-modified ceramides, and analyzed after click reaction with DIBO Alexa Fluor 488. Figure 6 shows emission spectra of liposome samples with different CHOL contents, normalized to the particle concentrations, as determined by the NTA.

These results show that liposome samples with a 50 vol% CHOL have lower emission intensities than those with 25 vol%. However, the incorporation of ω-N_3_-C_16_-Cer into C_16_-Cer-containing liposomes led to more intense signals than the incorporation of ω-N_3_-C_6_-Cer into C_6_-Cer-containing vesicles (Figure 5 and Figure 6). In addition, while the fluorescence signals stemming from clicked ω-N_3_-C_6_-Cer were proportional to the C_6_-ceramide content of the liposomes, this was less clear for ω-N_3_-C_16_-Cer- and C_16_-Cer-containing liposomes (Figure 5). Therefore, the incorporation of ω-N_3_-C_6_-Cer allowed liposomal ceramide content to be quantified, while the incorporation of ω-N_3_-C_16_-Cer offered a higher degree of sensitivity at the cost of poor quantification.

### 3.4. Ceramide-Containing Liposomes Maintain Splenocyte Viability

**Figure 7:**
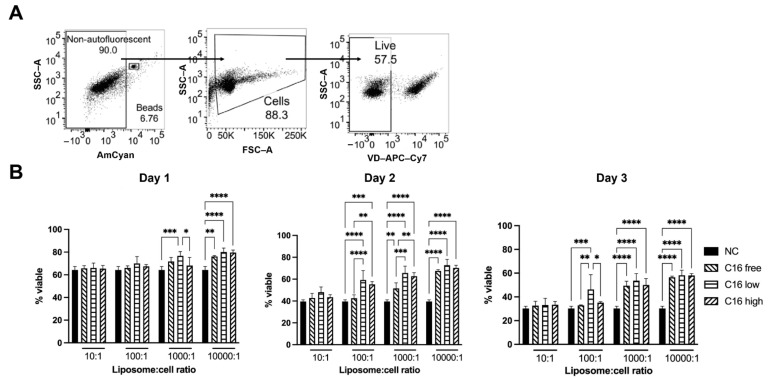
Mouse splenocytes were cultured for up to three days in the presence of different amounts of C_16_-Cer-free or C_16_-Cer-containing liposomes amounting to liposome: cell ratios of 10:1 to 10,000:1. (**A**) Viability and subpopulation composition were determined on a daily basis by flow cytometry using a viability dye (VD). (**B**) Means + SD of three independent experiments are shown. Two-way ANOVA followed by Tukey’s test. * *p* < 0.5, ** *p* < 0.01, *** *p* < 0.001, **** *p* < 0.0001. *p* < 0.05 was considered statistically significant.

Liposomes are widely used as nanocarriers for drug delivery including RNA-based vaccines such as BNT162b2 (Comirnaty ^®^) [32]. To investigate the impact of liposomes without and with high versus low content of C_16_-Cer, we co-cultured primary mouse splenocytes in the presence of different amounts of liposomes for up to three days (Figure 7). While we did not observe activation or proliferation of B and T cells as read out by the markers CD69, CD25, and Ki-67 (unpublished data) in the presence of liposomes, we observed a clearly beneficial effect on the viability of B and T cells as well as monocytes (Figure 7). Comparing the different liposomal preparations, C_16_-Cer-containing liposomes were slightly more efficient than Cer-free liposomes in maintaining splenocyte viability.

**Figure 8:**
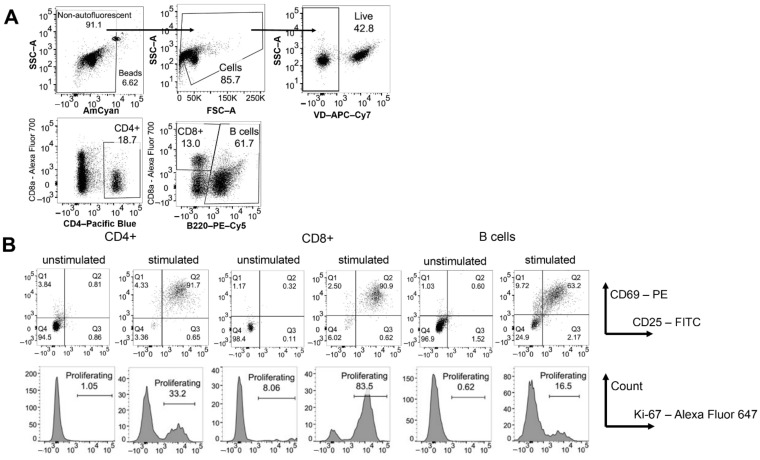
Mouse splenocytes were stimulated with Con A and cultured for two days. (**A**) Gating strategy to identify B cells, CD4^+^, and CD8^+^ T cells. (**B**) Lymphocyte activation was determined by analyzing CD69 and CD25 expression (upper row). Ki-67 expression was used to identify proliferating cells (lower row).

**Figure 9:**
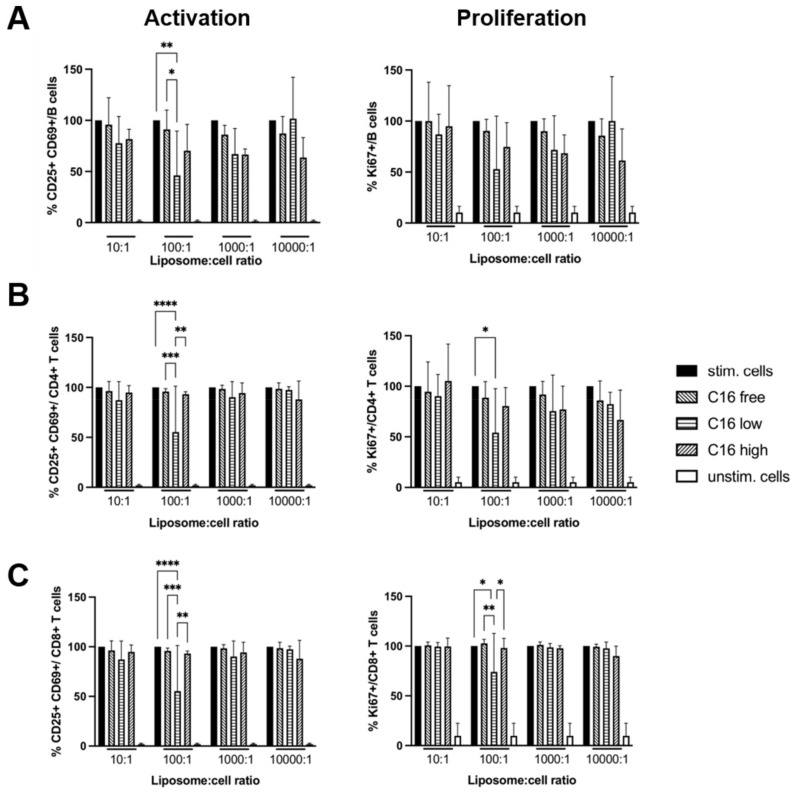
Summary graphs of results for (**A**) B cells, (**B**) CD4^+^ T cells, and (**C**) CD8^+^ T cells. Activated B and T cells were identified as CD25^+^ CD69^+^ (left column) and proliferating cells as Ki-67^+^ cells (right column). Values were normalized to stimulated cells in the absence of liposomes (=100%, black columns). Means + SD of *n* = 4 independent experiments. Two-way ANOVA followed by Tukey’s test. * *p* < 0.5, ** *p* < 0.01, *** *p* < 0.001, **** *p* < 0.0001. *p* < 0.05 was considered statistically significant. Comparisons to unstimulated cells rendered significant results throughout (*p* < 0.05) and are not shown for clarity.

The activation of B and T cells, particularly of T_reg_, greatly increases exosome release [25], which might provide a natural source of ‘external’ phospho- and sphingolipids similar to the liposomes (Figure 7). Therefore, we cultured mouse splenocytes in the presence of concanavalin A (Con A) to stimulate both B and T cells and followed the viability, activation, and proliferation of the cells for up to two days (Figure 8). Compared to unstimulated splenocytes, the addition of liposomes had less of an effect on the viability of cells. Only the C_16_-high liposomes exerted a dose-dependent effect on viability, i.e., high concentrations reduced the viability of splenocytes, while the lowest ratio of liposomes to cells increased the viability of splenocytes (data not shown). Regarding lymphocyte activation, B cell as well as CD4^+^ and CD8^+^ T cell activation were reduced in the presence of liposomes with a low content of C_16_-Cer (Figure 9A–C, left column). This was, however, only the case at a liposome to a cell ratio of 100:1. In line with reduced activation, the proliferation of CD4^+^ and CD8^+^ T cells was significantly reduced under these conditions and there was the same trend for B cells (Figure 9A–C, right column).

Ceramide-containing liposomes were thus superior to ceramide-free liposomes in maintaining the viability of splenocytes in vitro, especially at low liposome to cell ratios. Moreover, C_16_-low liposomes partially reduced lymphocyte activation and proliferation.

## 4. Discussion

Measuring the ceramide content of the cell membrane provides crucial insights into cellular activation and differentiation states. This is particularly true for lymphocytes which rely on sensing signals from the environment for mediating their function. Azide-functionalized ceramides offer the chance to assess ceramide content in the cell membrane of lymphocytes. However, so far, it has been unclear whether differences in the azide-functionalized ceramide incorporation into the membranes of T_reg_ and T_conv_ actually reflect differences in the ceramide content in the cell membrane of T_reg_ and T_conv_.

Liposomes constitute reductionist models of spherical lipid bilayers which we used to determine the contribution of the ceramide content of membranes to the incorporation of azide-functionalized ceramide. For this, we established a protocol to synthesize liposomes containing different amounts of ceramide. We could thus use liposomes with a high ceramide content (vol% ratio of 30:45:25 (C_6_/_16_-Cer:POPC:CHOL)) as membrane models of T_reg_, while liposomes with only half the ceramide content (vol% ratio of 15:35:50 (C_6_/_16_-Cer:POPC:CHOL)) served as a model for the plasma membrane of T_conv_ [6].

In general, liposomes with a higher ceramide content (30:45:25 vol% (C_6_/_16_-Cer:POPC:CHOL)) incorporated more clickable azide-functionalized ceramides than those with a lower ceramide content (15:35:50 vol% (C_6_/_16_-Cer:POPC:CHOL)) (Figure 4 and Figure 5). The differences between the degrees of post-incorporation into liposomes with high and low vol% ceramide were more pronounced for C_6_- than for C_16_-Cer, while the overall emission intensity was higher for C_16_- than for C_6_-Cer (Figure 4 and Figure 5). This suggests that the post-incorporation of azido-modified C_16_-Cer goes into saturation at lower C_16_-Cer than C_6_-Cer concentrations in membranes. The negative controls confirmed that no unspecific binding of the fluorescent dye to the lipid layer had occurred.

The investigation of CHOL influence on the post-insertion of ceramides shows that liposome samples with 50 vol% CHOL have lower emission intensities than those with 25 vol% (Figure 6). This indicates that vesicles with a higher CHOL content incorporated less clickable ceramides via post-incorporation. The same tendency was observed for the ceramide-free, C_6_-Cer- and C_16_-Cer-containing samples (unpublished data). As CHOL increases the packing density of the phospholipids, a high CHOL content causes a higher density of the phospholipid double layer. It is also known that CHOL is displaced from domains rich in ceramide [33]. If CHOL increases the packing density of the phospholipids around the ceramide microdomains, this influences the lateral diffusion of the ceramide molecules and prevents the incorporation of azido-modified ceramides. Ceramides prefer to accumulate in ceramide-rich membrane regions [10]. This hypothesis was confirmed by lower ceramide post-incorporation into ceramide-free liposomes into which the ceramide derivatives are incorporated more slowly and in lower quantities (Figure 5 and unpublished data). Samples with a ratio of 30:45:25 vol% (C_6_-Cer:POPC:CHOL) incorporated almost double the amount of azido-modified ceramides compared to vesicles with a ratio of 15:35:50 vol% (C_6_-Cer:POPC:CHOL) (Figure 5A). C_16_-Cer-modified samples incorporated relatively more azido-functionalized ceramide than C_6_-Cer-containing liposomes with an overproportioned incorporation into liposomes with low C_16_-Cer content (Figure 5B). A possible reason for this observation is that the liposomes were incubated with C_16_-Cer at 65 °C compared to 25 °C for C_6_-Cer to ensure post-incorporation of the C_16_-Cer. Generally, long-chain ceramides have a stronger influence on membrane fluidity than short-chain ceramides. The former can increase the membrane order and the phase transition temperature at which there is a shift from the gel to the liquid crystalline phase [34]. While both phases coexist in membranes, lipid monomers preferentially settle in the liquid crystalline phase due to its higher fluidity. Therefore, we incubated the liposomes with azido-modified C_16_-Cer at 65 °C instead of 25 °C. Additional experimentation is required to determine the conditions needed to achieve incorporation of C_16_-Cer into liposomes, and cells, in proportion to the ceramide content of the membrane. For the post-incorporation of ω-N_3_-C_6_-Cer, our data, however, clearly show that its degree reflected the Cer content of the liposomal membrane and thus might be suitable for quantification on a single-cell basis (Figure 1) [3,7]. To determine the precise relationship of membrane ceramide content and ω-N_3_-C_6_-Cer post-incorporation, more variations regarding the Cer content of the liposomes studied by us would have been necessary. However, only the two ratios of C_6_-Cer/C_16_-Cer:POPC:CHOL reported here resulted in stable liposomes with a diameter of about 100 nm suitable for further investigations.

A further limit of our study is that we did not compare the effect of ceramide on ceramide post-incorporation to that of other lipids, apart from, of course, CHOL. In particular, it will thus be interesting in future experiments to study how, e.g., saturated lipids such as dipalmitoyl glycerol, known to affect the biophysical properties of liposomal membranes [35], might impact ceramide post-incorporation.

Cellular membranes contain acidic phospholipids including phosphatidylserine and phosphatidic acid which, in viable cells, are primarily localized in the inner leaflet of the plasma membrane [1]. Therefore, we assume that they are not a crucial determinant for the incorporation of extracellularly added ceramides into the outer leaflet of the plasma membrane of T cells (Figure 1). Still, also for these lipid species, their impact on the capacity of liposomes to incorporate azido-modified ceramides should be analyzed in future experiments.

As liposomes are widely used for drug formulations including mRNA-based vaccine preparations [32], we studied the impact of the different liposomal preparations on splenocyte survival and activation in vitro. Independent of their ceramide content, we observed that the addition of liposomes enhanced splenocyte survival. In contrast to its apoptosis-inducing activity in solution, liposomal ceramide even enhanced the beneficial effect of the liposomes on cell survival (Figure 7) [36]. Currently, it is unclear how liposomes maintain viability of splenocytes in vitro. One possibility is that cells constantly take up exosomes/extracellular vesicles in vivo to replenish their lipid content. Organ isolation and the preparation of single-cell suspensions of splenocytes might cause a lack of exosomes/extracellular vesicles in in vitro cultures. The majority of splenocytes might be unable to compensate for this lack by increasing *de novo* lipid synthesis. In line with this notion, liposomes had less of an effect on the viability of stimulated concanavalin A (Con A) than on unstimulated splenocytes (Figure 9). The activation of B and T cells, particularly of T_reg_, greatly increases exosome release [25], which might compensate for the putative lack of exosomes/extracellular vesicles in the cultures of unstimulated splenocytes.

Ceramide-containing liposomes might thus be used in the future to enhance the yield and quality of difficult-to-culture cells, e.g., tumor-infiltrating lymphocytes used for cancer immunotherapy [37].

In contrast to the positive effect of liposomes, both with and without ceramide, on cell viability, B and T cell activation and proliferation were inhibited in the presence of liposomes with lower ceramide content and a liposome to cell ratio of 100:1. In addition, we noted that freshly prepared liposomes were strikingly more potent than liposomes stored at 4 °C for one week or more (unpublished data). Currently, it is unclear why we observed reduced T and B cell activation and proliferation only in the presence of a certain type of liposome and at a liposome to cell ratio of 100:1. One reason for the less sustained activation of B and T cells in the presence of ceramide-containing liposomes could be the fusion of liposomes and lymphocytes, leading to increases in ceramide content in the cell membrane of the T cells. For T cells, it has been shown that ceramide interferes with T cell receptor nanoclustering, which reduces the sensitivity of the T cells towards antigenic stimulation [35].

## 5. Conclusions

In summary, this study showed that ceramide-containing liposomes can be used as cell membrane models to test post-incorporation of ceramides into lipid bilayers. Regardless of the length of the ceramide chain, ceramide-containing vesicles incorporated more azide-functionalized ceramides than ceramide-free vesicles; in other words, a higher initial content of ceramides in the liposome membrane led to a higher post-incorporation capacity. This supports the assumption that the higher incorporation of clickable ceramide into T_reg_ than into T_conv_ actually reflected a higher ceramide content in the plasma membrane of T_reg_ than of T_conv_, where, indeed, clickable ceramides can be found in ex vivo analyses (Figure 1) [10,38]. For T cells whose biology and function heavily depend on the stimulation of cell surface receptors such as the T cell receptor and costimulatory molecules such as CD28, being able to quantitate the ceramide content of the cell membrane is crucial. This knowledge will help us obtain a better understanding of how the sphingolipid composition of the cell membrane influences T cell subsets and responses. Here, measuring the incorporation of clickable ceramides has clear advantages over the mass spectrometry of whole cell lysates, reflecting overall ceramide content.

## Figures and Tables

**Table 1:** Lipids and their amounts used for the preparation of stock solutions.

Lipid	Molecular Weight [g/mol]	Solvent	Lipid Concentration [mM]	Volume [mL]	Mass [mg]
POPC	760.08	CHCl_3_	5	50	190.0
CHOL	386.65	CHCl_3_	5	50	96.7
C_6_-Cer	397.64	CHCl_3_	5	50	99.4
C_16_-Cer	537.91	CHCl_3_	5	50	134.5
ω-N_3_-C_6_-Cer	438.66	CHCl_3_	5	50	109.7
ω-N_3_-C_6_-Cer	438.66	EtOH	25	0.840	12.2
ω-N_3_-C_16_-Cer	578.93	CHCl_3_	5	50	144.7
ω-N_3_-C_16_-Cer	578.93	EtOH	25	0.976	10.7

**Table 2:** Lipid compositions, particle sizes, and concentrations of liposomes with different ceramide contents. The hydrodynamic diameter and the PDI represent the average of 12 measurements. * particle size determined by dynamic light scattering (DLS); ** polydispersity index; *** mean size and liposome concentration determined by nanoparticle tracing analysis (NTA).

Lipid Composition	Volume Percentage Ratio [%]	Hydrodynamic Diameter * [nm]	PDI **	Liposome Size *** [nm]	Liposome Concentration *** [Particle/mL]
POPC:CHOL	75:25	126 ± 3	0.105	133	2.48 × 10^11^
C_6_-Cer:POPC:CHOL	15:35:50	132 ± 7	0.178	134	3.26 × 10^11^
C_16_-Cer:POPC:CHOL	15:35:50	130 ± 7	0.165	133	2.92 × 10^11^
C_6_-Cer:POPC:CHOL	30:45:25	110 ± 3	0.158	105	2.16 × 10^11^
C_16_-Cer:POPC:CHOL	30:45:25	111 ± 5	0.158	113	2.86 × 10^11^

**Table 3:** Liposome composition and concentrations.

Lipid Composition	Ceramide Mass per Unit Volume [mg/mL]
C_6_-Cer:POPC:CHOL	
30:45:25	2.79
15:35:50	2.595
C_16_-Cer:POPC:CHOL	
30:45:25	3.135
15:35:50	2.7

## Data Availability

Not applicable.

## Supplementary Materials

The following supporting information can be downloaded at: https://www.mdpi.com/article/10.3390/jfb13030111/s1, Scheme S1: Synthesis of the azido-modified ceramide probes ω-N_3_-C_6_-Cer (4) and ω-N_3_-C_16_-Cer (5); Figure S1: ^1^H-NMR spectrum (CDCl_3_, 400 MHz) of ω-N_3_-C_6_-Cer (4); Figure S2: ^13^C-NMR spectrum (CDCl_3_, 150 MHz) of ω-N_3_-C_6_-Cer (4); Figure S3: ^1^H-NMR spectrum (CDCl_3_, 400 MHz) of ω-N_3_-C_16_-Cer (5); Figure S4: ^13^C-NMR spectrum (CDCl_3_, 100 MHz) of ω-N_3_-C_16_-Cer (5); Figure S5: Mass spectrum (ESI^+^) of ω-N_3_-C_6_-Cer (4); Figure S6: Mass spectrum (ESI^+^) of ω-N_3_-C_16_-Cer (5).
