# Supplementary material for: Synthesis and Characterization of Ceramide-Containing Liposomes as Membrane Models for Different T Cell Subpopulations
Source: J Funct Biomater. 2022 Aug 2;13(3):111. doi: 10.3390/jfb13030111 (PMC9397063; doi:10.3390/jfb13030111)
Supplement: Supplementary file 1 [file jfb-13-00111-s001.zip › jfb-1787271-supplementary.pdf]

SUPPLEMENTAL INFORMATION:

Synthesis and characterization of ceramide-containing liposomes as membrane models for different T cell subpopulations

Sascha Eder [1], Claudia Hollmann [2°], Putri Mandasari [2°], Pia Wittmann [1,2], Fabian Schumacher [3], Burkhard Kleuser [3], Julian Fink [4], Jürgen Seibel [4], Jürgen Schneider-Schaulies [2], Christian Stigloher [5], Niklas Beyersdorf [2+] and Sofia Dembski [1,6+*]

1 Fraunhofer Institute for Silicate Research, ISC, Würzburg, Germany; eder.sascha@freenet.de; pia.wittmann@stud-mail.uni-wuerzburg.de; sofia.dembski@isc.fraunhofer.de

2 University of Würzburg, Institute for Virology and Immunobiology, Würzburg, Germany; claudia.hollmann@gmx.net; putri.mandasari@uni-wuerzburg.de; pia.wittmann@stud-mail.uni-wuerzburg.de; jss@vim.uni-wuerzburg.de; niklas.beyersdorf@vim.uni-wuerzburg.de

3 Freie Universität Berlin, Institute of Pharmacy, Berlin, Germany; burkhard.kleuser@fu-berlin.de; fabian.schumacher@fu-berlin.de

4 University of Würzburg, Institute of Organic Chemistry, Würzburg, Germany; julian.fink@uni-wuerzburg.de; seibel@chemie.uni-wuerzburg.de;

5 University of Würzburg, Imaging Core Facility of the Biocenter, Würzburg, Germany; christian.stigloher@uni-wuerzburg.de

6 Department Tissue Engineering and Regenerative Medicine, University Hospital, Würzburg, Germany; sofia.dembski@isc.fraunhofer.de

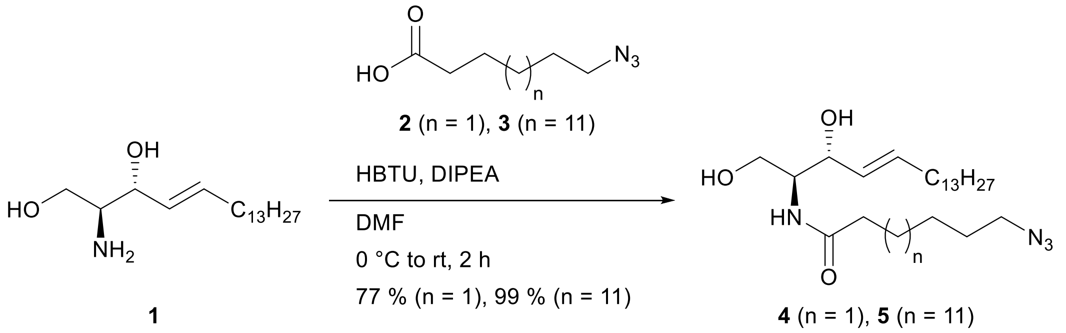

Scheme S1. Synthesis of the azido-modified ceramide probes ω-N_3-C_6-Cer (**4**) and ω-N_3-C_{16}-Cer (**5**).

NMR Spectra

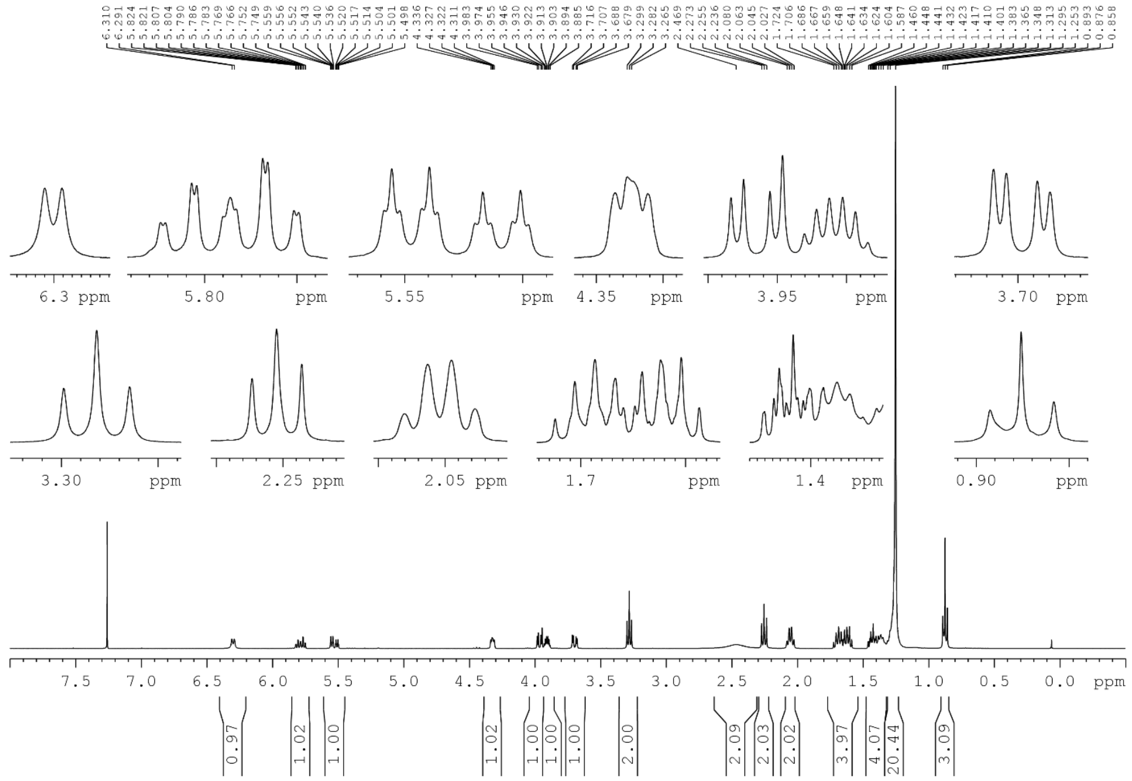

Figure S1. ^{1}H-NMR spectrum ($CDCl_3$, 400 MHz) of ω-N_3-C_6-Cer (**4**).

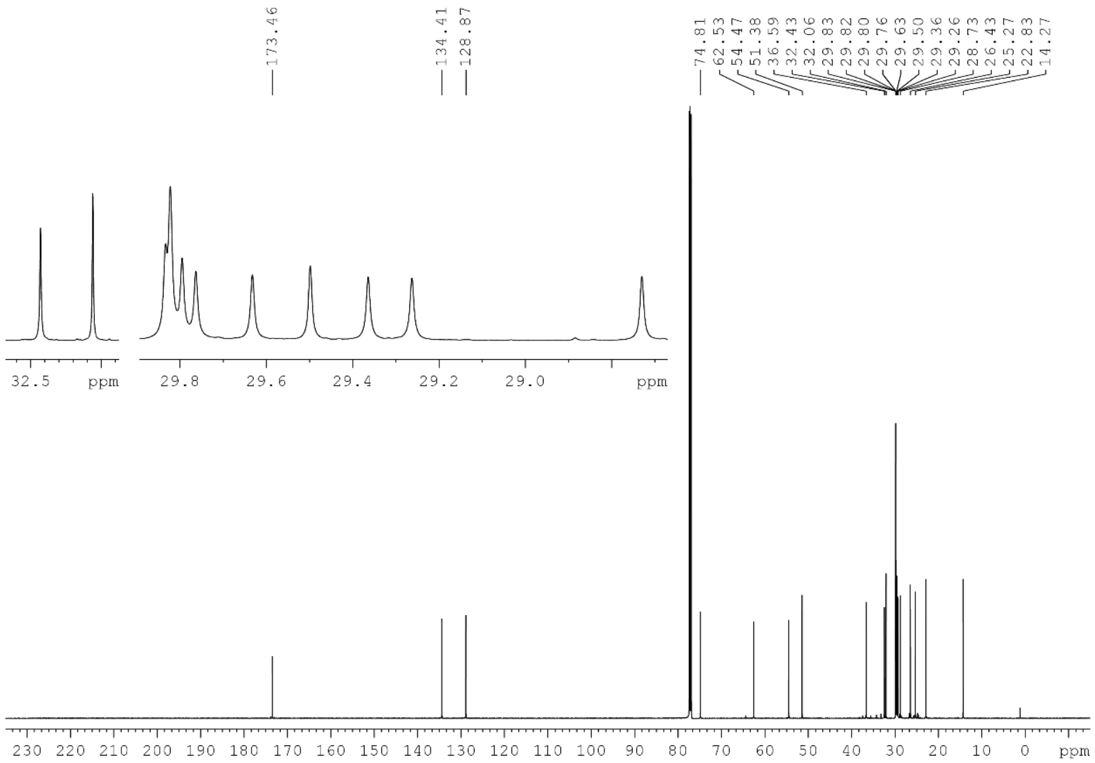

Figure S2. ^{13}C-NMR spectrum ($CDCl_3$, 150 MHz) of ω-N_3-C_6-Cer (**4**).

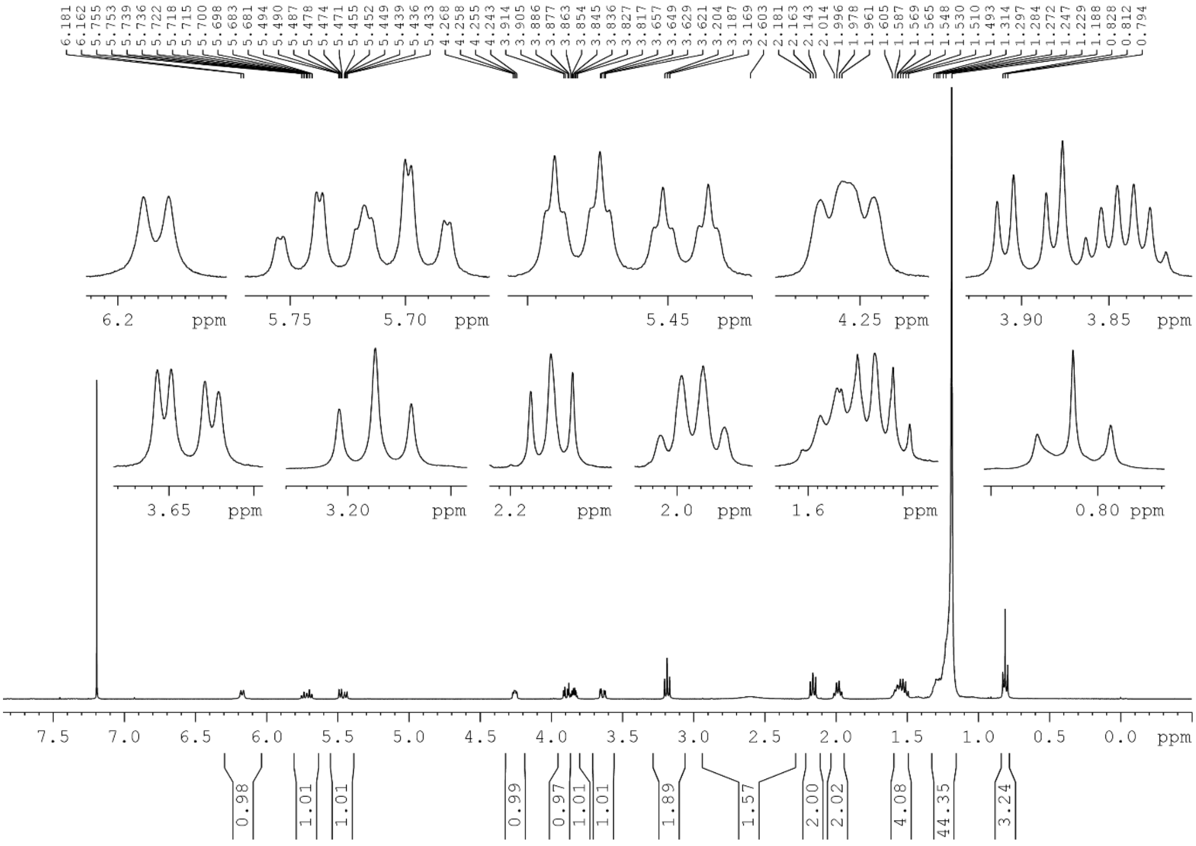

Figure S3. ^{1}H-NMR spectrum ($CDCl_3$, 400 MHz) of ω-N_3-C_{16}-Cer (**5**).

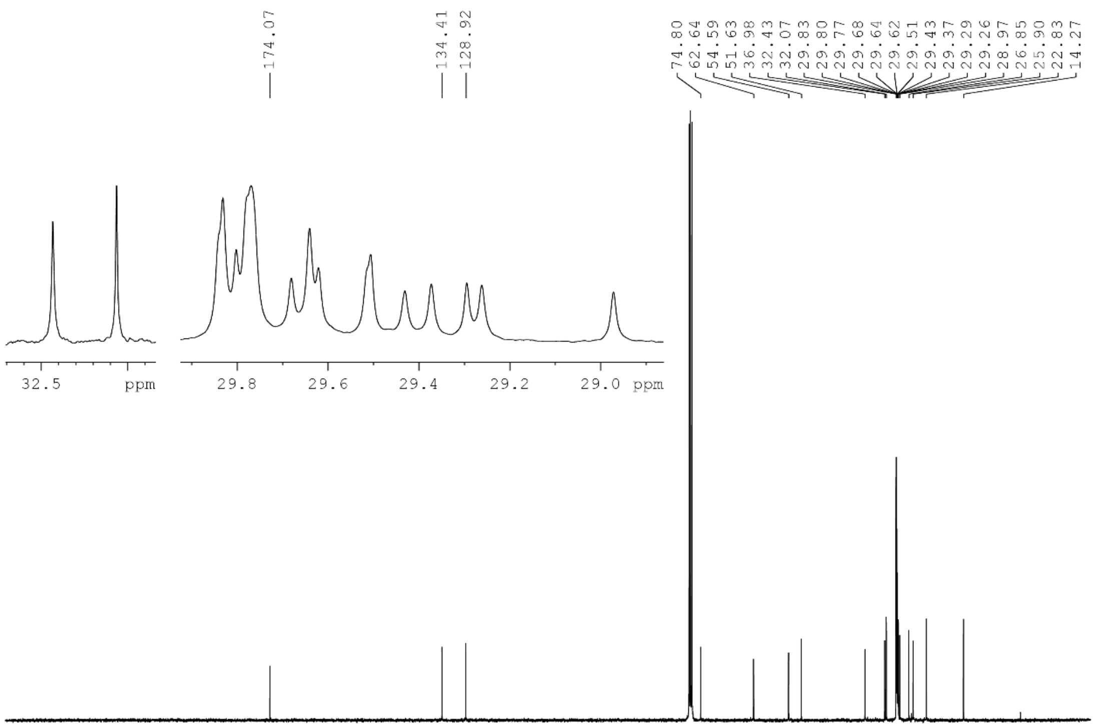

Figure S4. ^{13}C-NMR spectrum ($CDCl_3$, 100 MHz) of ω-N_3-C_{16}-Cer (**5**).

Mass Spectra

Analysis Info

Analysis Name	D:\Data\Spektren2018\2018_2707_SEI_JF158_3_01_4559.d	Acquisition Date	9/3/2018 3:17:49 PM
Method	automation_esi_tune_pos_low_ja.m	Operator	J.Adelmann
Sample Name	2018_2707_SEI_JF158	Instrument	micrOTOF-Q III 8228888.20516
Comment	Fink Julian<br>JF158<br>4 pmol/mL MeCN/CHCl3		

Acquisition Parameter

Source Type	ESI	Ion Polarity	Positive	Set Nebulizer	0.7 Bar
Focus	Not active	Set Funnel 1 RF	100.0 Vpp	Set Dry Heater	200 °C
Scan Begin	50 m/z	Set Funnel 2 RF	200.0 Vpp	Set Dry Gas	5.0 l/min
Scan End	3500 m/z	Set Hexapole RF	300.0 Vpp	Set Divert Valve	Source

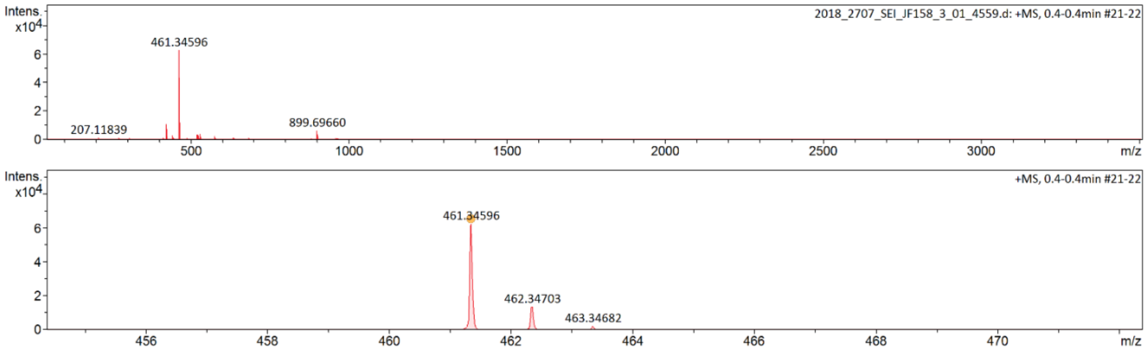

Meas. m/z	#	Ion Formula	m/z	err [ppm]	mSigma	# mSigma	Score	rdb	e⁻ Conf	N-Rule
461.34596	1	C24H46N4NaO3	461.34621	0.54	39.1	1	100.00	3.5	even	ok

Figure S5. Mass spectrum (ESI+) of ω-N_3-C_6-Cer (**4**).

Analysis Info

Analysis Name	D:\Data\Spektren2017\2018_0038_SEI_JF112_26_01_3166.d	Acquisition Date	1/9/2018 12:18:33 PM
Method	automation_esi_tune_pos_low_ja.m	Operator	J.Adelmann
Sample Name	2018_0038_SEI_JF112	Instrument	micrOTOF-Q III 8228888.20516
Comment	Fink Julian<br>JF112<br>6 pmol/ul in MeCN/CHCl3		

Acquisition Parameter

Source Type	ESI	Ion Polarity	Positive	Set Nebulizer	0.7 Bar
Focus	Not active	Set Funnel 1 RF	100.0 Vpp	Set Dry Heater	200 °C
Scan Begin	50 m/z	Set Funnel 2 RF	200.0 Vpp	Set Dry Gas	5.0 l/min
Scan End	3500 m/z	Set Hexapole RF	300.0 Vpp	Set Divert Valve	Source

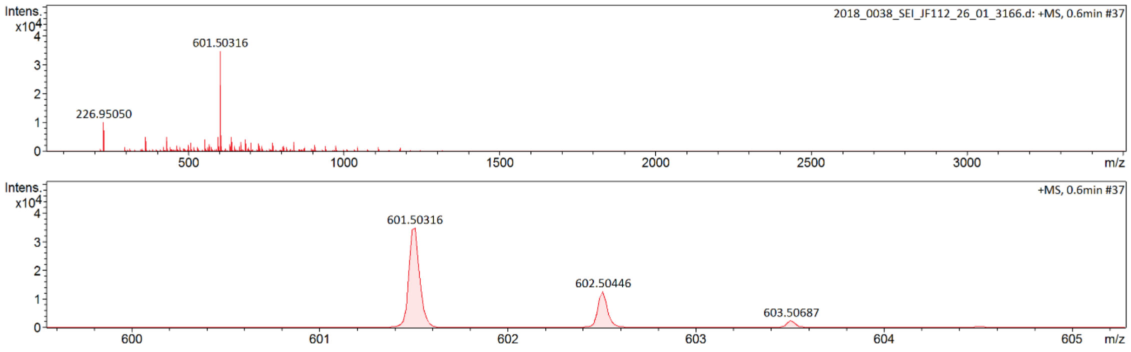

Meas. m/z	#	Ion Formula	m/z	err [ppm]	mSigma	# mSigma	Score	rdb	e⁻ Conf	N-Rule
601.50316	1	C34H66N4NaO3	601.50271	-0.75	15.2	1	100.00	3.5	even	ok

Figure S6. Mass spectrum (ESI+) of ω-N_3-C_{16}-Cer (**5**).
